# Draft Genome Sequence of the Plant Growth-Promoting Bacterium Pseudomonas pseudoalcaligenes KB-10

**DOI:** 10.1128/MRA.00311-21

**Published:** 2021-05-20

**Authors:** M. M. A. Khan, Jin Duan, Bernard R. Glick, Patrick M. Finnegan, Saleh A. Kabli, Saleh M. S. Al-Garni

**Affiliations:** a Department of Biological Sciences, King Abdulaziz University, Jeddah, Saudi Arabia; b Department of Biology, University of Waterloo, Waterloo, Ontario, Canada; c School of Biological Sciences, University of Western Australia, Perth, Western Australia, Australia; University of Arizona

## Abstract

Pseudomonas pseudoalcaligenes KB-10 can enhance salinity tolerance in coriander plants. We report a draft genome sequence of P. pseudoalcaligenes KB-10, comprising a 5,241,174-bp circular chromosome containing 4,921 genes, with a GC content of 62.97%.

## ANNOUNCEMENT

Plant growth-promoting bacteria (PGPB) can enhance plant growth and tolerance to various abiotic stresses including salinity (1). Here, we report the draft genome sequence of Pseudomonas pseudoalcaligenes strain KB-10. This bacterium was isolated from the rhizosphere of alfalfa (*Medicago sativa* L.) growing at the Agricultural Research Station of King Abdulaziz University located at Hada Al-Sham, Jeddah, Saudi Arabia (2). The strain was isolated using nutrient agar medium with an incubation period of 48 h at 30°C. The purified strain was stored in a 20% glycerol solution at −80°C for subsequent use. The strain was assessed for its ability to tolerate NaCl, to produce indole-3-acetic acid and 1-aminocyclopropane-1-carboxylate deaminase, and to solubilize tricalcium phosphate. The strain was able to tolerate up to 5% NaCl and to produce indole-3-acetic acid (2). A pot experiment revealed that the isolated strain augmented plant growth and salinity tolerance in coriander (Coriandrum sativum L.) (2).

For genomic DNA isolation, a single colony of the strain was grown in nutrient broth overnight at 30°C. The genomic DNA was extracted using a genomic DNA purification kit (MGmed, South Korea) according to the manufacturer's instructions. A DNA library was generated using the TruSeq DNA Nano sample preparation kit (Illumina, Inc., CA, USA), and then paired-end (2 × 100-bp) sequencing was performed using the HiSeq 4000 platform (Illumina, Inc.) at Macrogen (South Korea), generating a total of 1,702,568,312 bp from 16,857,112 reads. After sequencing, FastQC version 0.11.6 (3) was used in order to check the read quality. In order to reduce biases in the analysis, an in-house script was used to filter reads if less than 90% of the bases that made up the read had a score below Q20. Trimmomatic version 0.36 (4) was used to remove the adapter sequences. After filtering, genome sequencing yielded 1,094,661,536 bases from 11,050,922 reads. For *de novo* assembly, a genome survey was performed with Jellyfish version 1.1.11 (5) to first estimate the genome size of the sample. In this step, 17, 21, and 25 k-mers were used for calculation of the estimated genome size, and the k-mer plot was expressed with GenomeScope (6). After the genome survey, the initial *de novo* genome assembly was performed with various k-mer values with SOAPdenovo2 version 2.04 (7), and the gap-closing process was performed with GapCloser version 1.112-r6, which resulted in 28 contigs ranging from 1,135 bp to 1,079,592 bp. Prokka version 1.12b was used for gene prediction and annotation (8). The assembled sequences were also used for searches against the GenBank nonredundant, UniProt, GO, InterPro, Pfam, conserved domain (CD), TIGRFAM, and eggNOG databases using BLAST+ version 2.6.0 for additional annotation (9). Default parameters were used for all software unless otherwise mentioned.

The draft genome of P. pseudoalcaligenes KB-10 contains 5,241,174 bp with a GC content of 62.97%. A total of 4,921 genes were predicted, with 4,852 of the genes corresponding to protein-coding sequences (CDSs). A total of 65 tRNA, 1 transfer-messenger RNA, and 3 rRNA genes were also predicted. The *hisC1* gene encoding histidinol phosphate aminotransferase, which is involved in the indole-3-pyruvic acid pathway for the synthesis of indoleacetic acid, was identified.

### Data availability.

The draft genome sequence of P. pseudoalcaligenes KB-10 has been deposited in the NCBI GenBank database under the accession number QYYB00000000. The raw sequencing data have been deposited under accession numbers PRJNA483993 (BioProject) and SRX10436105 (Sequence Read Archive [SRA]). The version described in this paper is the first version.
